# Improved cardiovascular diagnostic accuracy by pocket size imaging device in non-cardiologic outpatients: the NaUSiCa (Naples Ultrasound Stethoscope in Cardiology) study

**DOI:** 10.1186/1476-7120-8-51

**Published:** 2010-11-26

**Authors:** Maurizio Galderisi, Alessandro Santoro, Marco Versiero, Vincenzo Schiano Lomoriello, Roberta Esposito, Rosa Raia, Francesca Farina, Pier Luigi Schiattarella, Manuela Bonito, Marinella Olibet, Giovanni de Simone

**Affiliations:** 1 Department of Clinical and Experimental Medicine, Federico II University Hospital, Naples, Italy

## Abstract

Miniaturization has evolved in the creation of a pocket-size imaging device which can be utilized as an ultrasound stethoscope. This study assessed the additional diagnostic power of pocket size device by both experts operators and trainees in comparison with physical examination and its appropriateness of use in comparison with standard echo machine in a non-cardiologic population.

Three hundred four consecutive non cardiologic outpatients underwent a sequential assessment including physical examination, pocket size imaging device and standard Doppler-echo exam. Pocket size device was used by both expert operators and trainees (who received specific training before the beginning of the study). All the operators were requested to give only visual, qualitative insights on specific issues. All standard Doppler-echo exams were performed by expert operators.

One hundred two pocket size device exams were performed by experts and two hundred two by trainees. The time duration of the pocket size device exam was 304 ± 117 sec. Diagnosis of cardiac abnormalities was made in 38.2% of cases by physical examination and in 69.7% of cases by physical examination + pocket size device (additional diagnostic power = 31.5%, p < 0.0001). The overall K between pocket size device and standard Doppler-echo was 0.67 in the pooled population (0.84 by experts and 0.58 by trainees). K was suboptimal for trainees in the eyeball evaluation of ejection fraction, left atrial dilation and right ventricular dilation. Overall sensitivity was 91% and specificity 76%. Sensitivity and specificity were lower in trainees than in experts.

In conclusion, pocket size device showed a relevant additional diagnostic value in comparison with physical examination. Sensitivity and specificity were good in experts and suboptimal in trainees. Specificity was particularly influenced by the level of experience. Training programs are needed for pocket size device users.

## Introduction

The dream of hand-held echocardiography first materialized during the 1970 s. Since then its progress has continued with the development of several kinds of portable ultrasound machines which are currently used in clinical practice. Several of these instrumentations serve as adequate application tools and in many instances innovative technology makes them very similar to standard echocardiographic machines; other portable machines have only basic diagnostic tools but excellent transportability and low cost [1]. In order to clarify the differences and peculiarities of hand-held echocardiography, in 2002 the American Society of Echocardiography differentiated complete portable instrumentations from battery-operated machines of weight lower than 6 lb (2-5-2.7 Kg) which do not fulfil the criteria for a comprehensive ultrasound assessment of the heart [2].

Very recently, an extreme miniaturization of echo machines has evolved with the creation of a pocket-size imaging device (PSID), small and light enough to fit in the hand. This instrument provides black and white anatomic and colour-flow images in real time. Conceptually, the cardiologic applications of PSID include the possibility of using it in coronary and emergency care units, for a first cardiac approach in ambulances, for screening programs in communities, and during cardiology consultations inside or outside the hospital. The latter application highlights the possible use of this instrument as a completion of the traditional physical exam (PE), to add incremental information and, when needed, to appropriately refer patients to a standard Doppler echocardiographic examination.

In this view, PSID might be utilized by clinicians as a true ultrasound stethoscope. However, the diagnostic accuracy of this device has not yet been tested in comparison with full-application ultrasound machines (as a gold standard) and in relation to the level of experience of the users, another relevant factor in the evaluation of the potential spread of its use. Accordingly, the present study was designed to assess in a sample of non-cardiologic patients with intermediate to high risk cardiac involvement: 1. The incremental diagnostic value of PSID to PE by both expert operators and trainees, 2. The diagnostic accuracy of PSID in identifying a limited number of parameters determinable by this new device in comparison with standard Doppler echocardiography and also in relation to the level of ultrasound experience (comparison between experts operators and trainees).

## Methods

The study population comprised all the consecutive outpatients who were referred from the Department of Endocrinology and Oncology (endocrinology disorders, evaluations for oncological and hematological pathologies before, during and/or after chemotherapy) for a cardiac consultation between November 2009 and July 2010 to the Cardioangiology Unit of Federico II University Hospital of Naples. The indications for cardiac consultation were represented by a routine pre-therapy evaluation or during/after therapy evaluation because of the onset of cardiac symptoms for oncological and hematological patients and by the assessment of cardiac risk in endocrinological patients. Cardiac ultrasound assessment was justified by an intermediate (e.g., thyroid and surrenal disorders) to a high risk (e.g., oncology patients undergoing chemotherapy and/or radiotherapy). All subjects gave written informed consent and the study was approved by the Institutional Ethical Committee.

All the patients underwent a sequential assessment including PE, PSID and standard Doppler echocardiographic exams during the same morning. To be eligible for this study, patients were required to have an acceptable ultrasound imaging by PSID. Exclusion criteria included clinically overt heart failure, a history of coronary artery disease and/or previous myocardial infarction, recognized valvular heart disease, or primary cardiomyopathies. Among the initial referred population of 321 outpatients, 304 (94.7%) were considered eligible for the study. Seventeen patients were excluded: 4 because of inadequate imaging quality by PSID, 4 because of clinical overt heart failure, 4 because of previous myocardial infarction, 3 because of severe valvular heart disease (2 aortic valve stenosis and 1 mitral regurgitation) and 2 because of primary cardiomyopathy (1 dilated and 1 hypertrophic cardiomyopathy).

The PE included exploration of heart rhythm and cardiac murmurs, jugular vein inspection (detection of jugular venous distention) and liver palpation (size detection), thorax auscultation and detection of ankle oedema.

Cardiac ultrasound examination was performed using PSID (*Vscan*, Horten, Norway), a light pocket-size instrument (unit + probe = 390 g) which provides black and white two-dimensional (2-D) and colour-coded blood flow images (fixed colour-box size and fixed pulse-repetition frequency) in real time and is connected to a broad-bandwith, phased array probe (1.7 to 3.8 MHz). The flow sector represents blood flow within an angle of 30 degrees. Images and videos (automatic autocycle without the need of ECG) can be stored in examination folders, recalled via a gallery function and transferred to PC or USB through a docking station. Two different machine settings are available: the first for cardiac and thoracic application, the second for abdominal, obstetric and gynecological applications.

In the present study we used the cardiac setting including a complete 2-D scanning (parasternal, apical and subcostal views) and colour-flow images of the cardiac valves. At the end of the study, cuff blood pressure (mean of 3 measurements) was estimated by a physician, blinded to the examination. PSID was used by both expert operators (more than 3 years' experience in cardiac ultrasound) and trainees (= residents of internal medicine who received specific training before the beginning of the study). The training program included 15 hours of instruction on basic principles of cardiac ultrasounds (mainly cardiac anatomy by standard views and echocardiographic imaging optimization) and 3 months (3 times per week, 12 examinations per day, totalling 145-150 examination) of handling and visual interpretation of PSID examinations with exclusively visual judgment of limited parameters (LV dilation and ejection fraction, LA dilation, RV dilation, valve calcification, pericardial and pleural effusion, valve calcifications, valve regurgitations, inferior vena cava size and reactivity, identification of ULC). All the operators were requested to give only visual, qualitative insights on specific pathologic issues: left ventricular (LV) dilation, LV wall hypertrophy, reduction of LV function (visual ejection fraction), right ventricular (RV) dilation, valve calcification, pericardial effusion, pleural effusion, ultra-lung comet (ULC) as a sign of interstitial lung accumulation, dilation + reduced inspiratory reactivity of inferior vena cava (IVC). Operators were additionally requested to identify more than trivial mitral regurgitation, aortic regurgitation and tricuspid regurgitation. The time duration of each PSID exam was calculated (in seconds) for each individual examination. Of note, the same operator performed PE and PSID exam.

Standard transthoracic full-featured Doppler echocardiographic examinations were performed by expert operators, blinded to the PSID results, with a Vivid Seven ultrasound scanner (GE, Milwaukee, WI, USA) using a 2.5 transducer with harmonic capability, according to the standards of our laboratory [3]. Quantification of chambers and flow hemodynamics were performed according to the standard methods [4-8]. In particular; LV wall thickness (posterior or septal wall) ≥ 1.1 cm was considered representative of LV wall hypertrophy, LV end-.diastolic volume ≥ 76 mL/m^2 ^of LV dilation, EF ≤ 55% of reduced LV systolic function, LA volume ≥ 34 ml/m^2 ^of LA dilation, basal RV diameter ≥ 2.9 cm + RV base-to-apex length ≥ 8.0 cm of RV dilation, IVC diameter > 1.7 cm with inspiratory collapse < 50% of increased right atrial pressure. A vena contracta ≥ 3 mm was considered significant for both mitral and aortic valve regurgitation. A retrograde tricuspid gradient ≥ 25 mmHg was considered representative of significant tricuspid regurgitation. Full-featured Doppler echo examination was taken into account as the gold standard for comparison.

### Statistical analyses

Statistical analysis was performed by SPSS package, release 12 (SPSS Inc, Chicago, Illinois, USA). Data are presented as mean value ± SD. Descriptive statistics were obtained by one-factor ANOVA (post-hoc analysis by Bonferroni test) and χ2 distribution with computation of exact p value by the Monte Carlo method. Sensitivity and specificity were calculated according to standard methods in the pooled population and compared between expert operators and trainees. Differences in the prevalence of echo findings between expert operators and trainees were compared using the Fisher exact test. The null hypothesis was rejected at 2-tailed p < 0.05.

## Results

The clinical characteristics of the study population are summarized in Table 1. The list of extra-cardiac diseases for which the cardiac consultation was requested is reported in Table 2.

**Table 1:** Characteristics of the Study Population

Characteristics	Mean ± SD	Mean ± SD	Mean ± SD
	Overall population	Female population	Male population
Total number	304	149	155
Age (years)	54.5 ± 17.7	53.3 ± 17.3	55.1 ± 18.1
BMI (Kg/m2)	26.6 ± 5.4	27.0 ± 6.4	26.2 ± 4.3
Systolic BP (mmHg)	129.4 ± 16.5	128.4 ± 16.6	130.3 ± 16.4
Diastolic BP (mmHg)	78.2 ± 9.9	78.7 ± 10.3	77.6 ± 9.4
Mean BP (mmHg)	95.2 ± 10.6	95.3 ± 11.1	95.2 ± 10.2

BP = Blood pressure, BMI = Body mass index

**Table 2:** List of extra-cardiac diseases referred for cardiologic consultation.

Referral Division	Disease	Number
Oncology (n = 153)	Breast cancer	27
	Lung cancer	25
	Colon-rectum cancer	20
	Gastric cancer	18
	Prostate cancer	14
	Liver cancer	12
	Bladder cancer	10
	Ovary cancer	8
	Uterus cancer	7
	Renal cancer	5
	Thymoma	4
	Pancreas cancer	3
Hematology (n = 70)	Chronic lymphocytic leukemia	16
	Acute myeloid leukemia	14
	Chronic myeloid leukemia	10
	Acute lymphocytic leukemia	9
	Non Hodgkin lymphoma	9
	Hodgkin lymphoma	6
	Multiple Myeloma	6
Endocrinology (n = 81)	Hypothyroidism	21
	Cushing	15
	Prolactinoma	14
	Acromegaly	12
	Primary Hyperaldosteronism	7
	GH Deficit	7
	Surrenal incidentaloma	5

One hundred two PSID exams (33.6%) were performed by expert operators and 202 (66.4%) by trainees. The time duration of PSID exams was 304 ± 117 sec in the pooled population (318 ± 139 sec in expert operators and 297 ± 104 sec in trainees, p = 0.137, NS).

**Figure 1:**
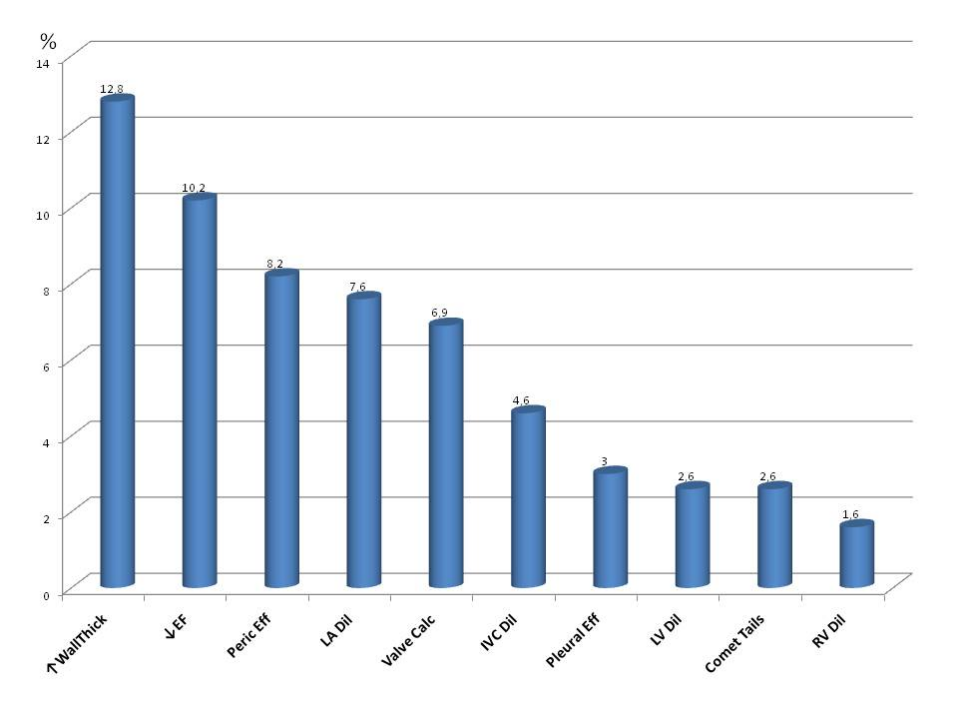
**Prevalence of cardiac abnormalities not detectable by PE and diagnosed by PSID in the overall population**.

**Figure 2:**
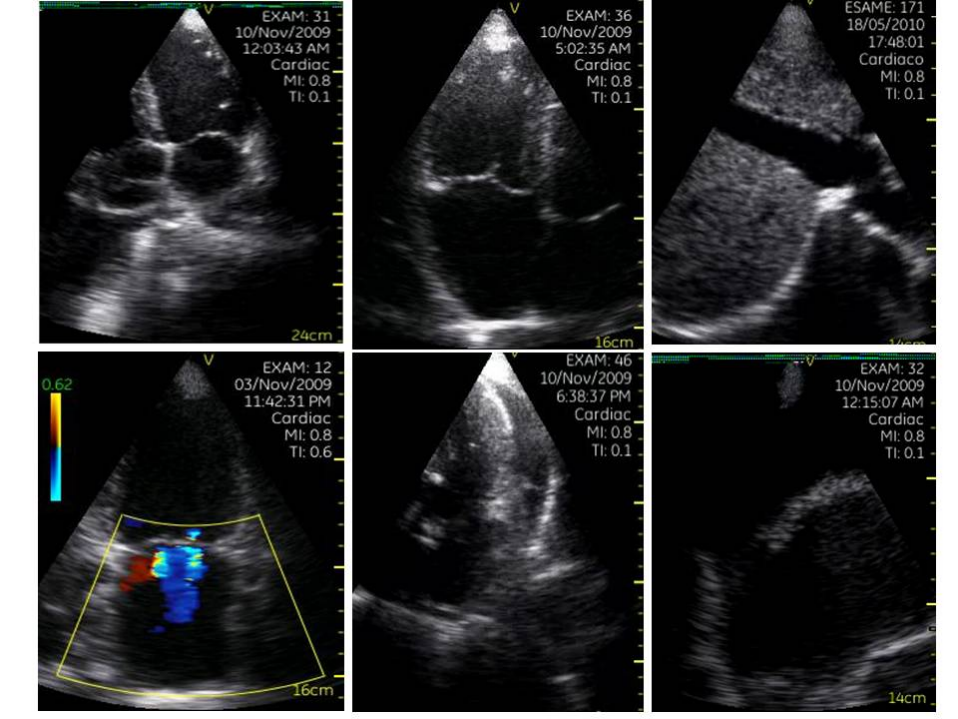
**Sample of abnormal findings detected by PSID in the study population**. In the first line (from left to right): enlarged left ventricle with depressed EF, dilated right ventricle and dilated IVC which also presents reduced respiratory reactivity. In the second line: mitral regurgitation (double jet), pericardial effusion and pleural effusion.

Diagnosis of cardiac abnormalities was made in 38.2% (116/304) via a simple PE and in 69.7% (212/304) through a combination of PE + PSID, with an additional diagnostic power of 31.5% (p < 0.0001). This difference remained significant even when analyzing separately the prevalence of cardiac abnormalities detected by experts (PE = 38.2% [39/102) vs PE + PSID = 73.5% [75/102], +35.3%, p < 0.0001) and trainees (PE = 38.1% [77/202) vs PE + PSID = 67.8% [137/202], +29.7%, p < 0.0001). Figure 1 shows the rate of cardiac abnormalities detected by PSID but not by PE. Figure 2 displays the imaging of some abnormalities detected by PSID.

Table 3 summarizes the concordance (kappa statistic for agreement) between the ultrasound examination performed by PSID and standard Doppler-echo as the gold standard. The overall K was 0.67 in the pooled population, as high as 0.84 for PSID exams performed by expert operators and 0.58 for trainees. As shown in Table 3 the concordance was maximal for valve calcification, pericardial and pleural effusion and ULC while it was relatively low for detecting LA dilation and RV dilation. The concordance of mitral regurgitation detection was lower in trainees than in expert operators.

**Table 3:** Concordance of the main findings between PSID and standard echo machine.

Pathologic Finding	K Overall (n = 304)	K Experts (n = 102)	K Beginners (n = 202)
Overall	0.67	0.84	0.58
↓ LV EF	0.89	0.91	0.87
↑ wall thickness	0.9	0.91	0.88
LA dilation	0.77	0.88	0.68
AO root dilation	0.95	1	0.91
RV dilation	0.87	0.9	0.81
IVC Dilation	0.96	1	0.79
Valve calcification	1	1	1
Pericardial effusion	1	1	1
Pleural effusion	1	1	1
ULC	0.94	1	0.91
MR	0.9	0.95	0.87
AR	0.94	1,00	0.91
TR	0.93	0.95	0.92

Comparison K Experts vs. Beginners by Mantel-Haenszel test

AO = Aortic, AR = Aortic regurgitation, EF = Ejection fraction, IVC = Inferior vena cava, LV = Left ventricular, MR = Mitral regurgitation, RV = Right ventricular, TR = Tricuspid regurgitation, ULC = Ultrasound lung comets

**Figure 3:**
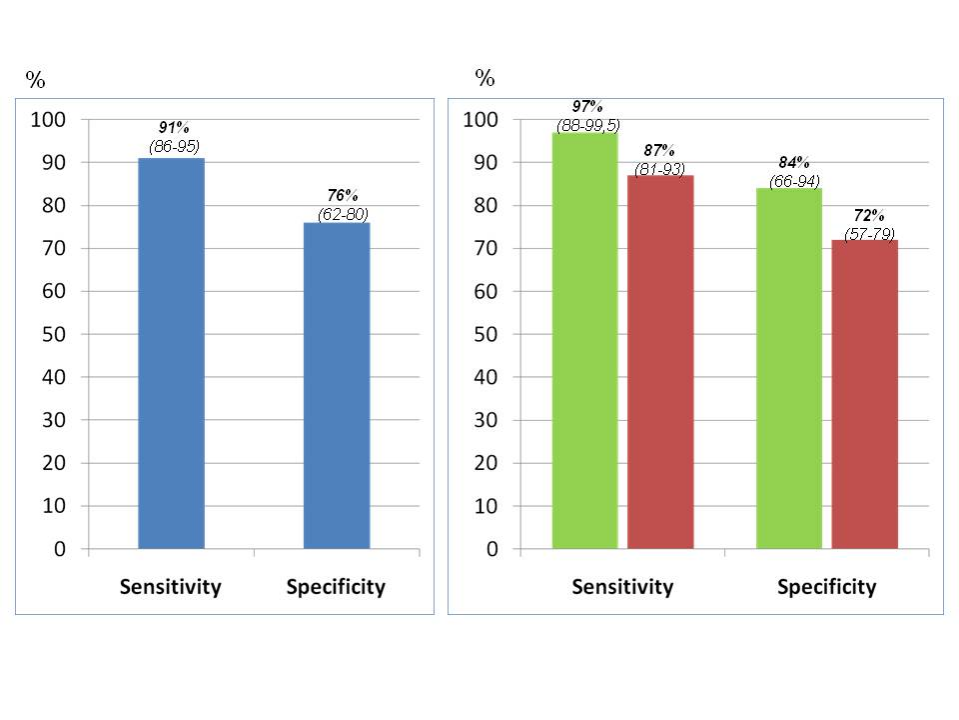
**Sensitivity and specificity (with 95% confidence intervals) of PSID in the overall population (left panel) and comparison of experts and trainees (right panel)**. Standard Doppler echo is the reference gold standard.

Figure 3 displays sensitivity and specificity in the overall population (91% and 76% respectively) and separately in experts and trainees. Sensitivity and, more particularly, specificity were lower in trainees than in experts due to the higher proportion of false positive results (10% in trainees vs 5% in expert operators) (data not shown in the Figure).

## Discussion

The present study demonstrates that PSID is useful in the detection of cardiac abnormalities and adds clinical information to PE in a population of patients referred for cardiac consultation, increasing the traditional consultation by only about 5 minutes. The simple visual assessment performed by PSID has a good concordance with standard Doppler echocardiographic examination and good diagnostic accuracy, which is higher in expert operators and lower in trainees.

PE has recognized limitations in subclinical cardiac diseases [9,10]. Noisy situations and uncomfortable thoracic conformations associated with concomitant diseases (e.g., obesity or emphysema) can further reduce the accuracy of PE. Accordingly, a novel clinical approach including the support of hand-held echocardiography to PE was proposed at the beginning of 2000 [11] and subsequently realized using a wide variety of hand-held echocardiographic machines. The combination of hand-held echocardiography + PE improves diagnostic sensitivity and reduces the rate of incorrect diagnosis (29% vs. 59% compared to the sole PE) [12]. Hand-held echocardiography has been shown to be useful in the detection of depressed LV systolic function [13], mitral valve prolapse [14], LV hypertrophy [15], ULC [16], aortic root dilation [17] and abdominal aorta aneurysms [18]. Of note, visual EF has been demonstrated to correlate closely with quantitative EF estimated by 3 D echocardiography [19]. The diagnostic accuracy of hand-held echocardiography can be affected by the type of referral in question, it being higher in patients referred for arterial hypertension or palpitations and lower in those who presented dyspnea or chest pain [20]. In a volunteer screening performed in the main squares of Naples and Siena in unselected people, we found subclinical cardiac abnormalities in 13.5% of the screened subjects (51/377) by using hand-held echocardiographic machines [21]. This type of screening might help, therefore, to detect unknown, asymptomatic cardiovascular disease and favor prevention programs.

The present study extends these findings to a population of non-primary cardiac outpatients by combining PE with visual PSID assessment and documents the clinical value of this instrumentation in helping diagnosis of a variety of cardiac abnormalities, not detectable by the simple PE. This is the first study to apply an exclusive visual, clear-cut assessment of a large variety of cardiac abnormalities by using cardiac ultrasound, an approach widely used in the clinical setting. We identified cardiac abnormalities in 38.2% of the assessed patients by PE and in 69.7% by the combination of PE + PSID, with a "theoretical" additional diagnostic power of 31.5%. By analyzing the additional diagnostic power of PSID according to the level of experience, this remained evident not only in expert operators (35.3%) but also in trainees (29.7%).

The diagnostic accuracy of the combination PE + PSID assessed by standard Doppler echocardiography as the gold standard was generally good, and even excellent for some specific abnormalities. The analysis of concordance highlighted the role of experience in echocardiography. Some findings (aortic root dilation, valve calcifications, pericardial effusion, pleural effusion, ULC, significant aortic valve regurgitation) had a very high concordance for both experts and trainees. Also the concordance of increased wall thickness and significant tricuspid regurgitation remained sufficiently high, whereas it was suboptimal for trainees in the eyeball evaluation of EF, LA dilation, RV dilation, IVC dilation and mitral regurgitation. The need for a learning curve is expected when assessing eyeball LV systolic function and both chamber and vessel size while the fixed colour-box size and the fixed pulse-repetition frequency of PSID can have limited estimation of the mitral regurgitation degree, currently difficult even when using standardized quantitative assessment [8].

The assessment of diagnostic accuracy (versus standard Doppler echo assessment) provided further information. In the overall population, sensitivity was very high (91%) while suboptimal specificity (76%) was justified by a certain amount of false positive results. When the assessment was performed according to the level of experience, both sensitivity and specificity appeared highly satisfactory for expert operators (97% and 84% respectively). Sensitivity remained good (87%) in trainees who showed, however, a relatively low specificity (72%). These results are in contrast with the findings of De Cara et al [22], who used a graded visual judgment (0 = trace, 1 = mild, 2 = moderate, 3 = severe) using a first generation hand-held device (Optigo, Philips); they found overall moderate sensitivity and high specificity whereas the comparison between expert operators and non-expert residents showed a significantly lower positive predictive value but similar negative predictive value in studies performed by the residents. Differences between the two studies account for the technical differences of the two machines used, the visual judgment of cardiac abnormalities - clear-cut in our experience and graded in the De Cara study, the population selection - primary non-cardiac patients and patients with established cardiac diseases respectively, and the level of experience of the trainees (background of 145-150 and 20 examinations performed respectively). In general, the findings of our study suggest a trend towards an overestimation of echo abnormalities in the trainees when using PSID.

**Figure 4:**
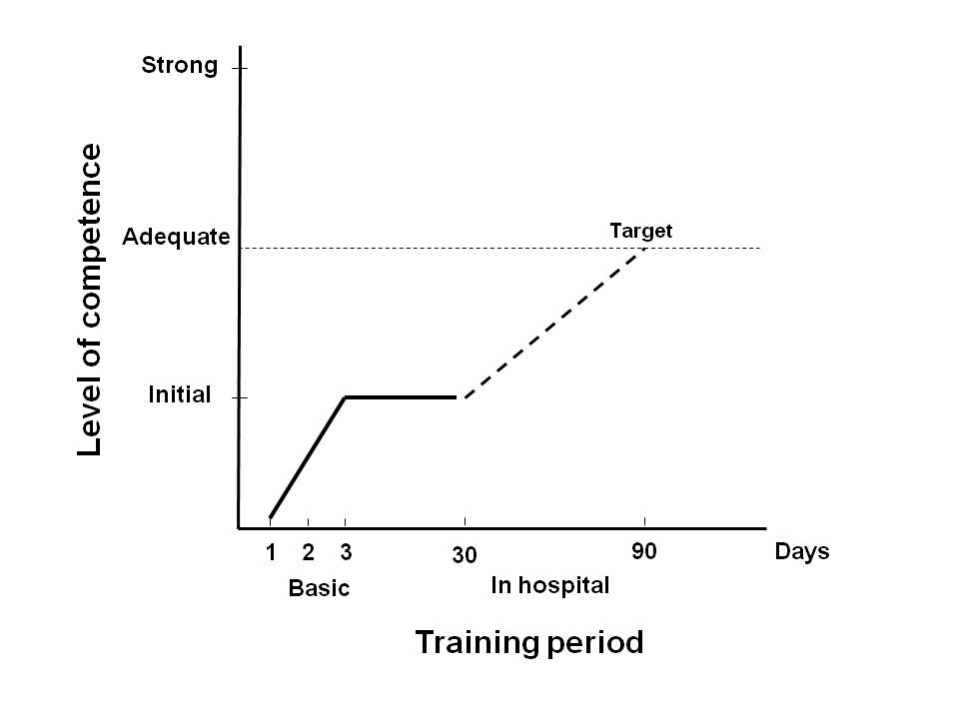
**Proposal of a training program model for PSID users**. A short period of 3 days basic teaching (ultrasound physics and biological effects, normal and pathologic cardiovascular anatomy, normal and pathologic blood flow dynamics) should be followed, after a free interval, by an in-hospital period (60 days, 3 days per week) for highly motivated operators (direct scan of standard echo views by PSID and visual assessment of at least 150 exams by PSID), in order to achieve the target level of competence.

Insights about the need for adequate competence level for the users of PSID rise from the present study. Expert operators do not need any training to adequately use PSID, whereas some training shall be recommended for operators without experience in cardiac ultrasound. A previous study showed an optimal concordance of results between cardiologic training of only 6 weeks and a "faculty" of expert sonographers for assessing LV systolic function by hand-held echocardiography [23]. Such short training is not advisable for an extensive PSID assessment including the detection of multiple abnormalities as in our study. Both the American Society of Echocardiography and the European Association of Echocardiography require basic knowledge from any person involved in performing or reading echocardiograms [24,25]. Several of these requirements should be encouraged also for PSID users and include the knowledge of ultrasound physics and biological effects, normal and pathologic cardiovascular anatomy, normal and pathologic blood flow dynamics. This part of the training should be followed by subsequent in-hospital training including direct scanning, with the performance of the standard echocardiographic views by PSID, and visual assessment of normal and abnormal findings, for a total amount of at least 150 PSID exams. We suggest that particular care should be taken in distinguishing 10 mean findings (Table 4). Figure 4 summarizes a model of a training program proposed for PSID users.

**Table 4:** The main 10 items to be searched by visual assessment with PSID

	Findings
1.	LV Ejection fraction

2,	Wall thickness

3.	Valve calcification

4.	Pericardial effusion

5.	Pleural effusion

6.	Ultra-lung comet tails

7.	RV dilation

8.	IVC dilation and reactivity

9.	Mitral regurgitation

10.	Tricuspid regurgitation

IVC = inferior vena cava, LV = Left ventricular, RV = Right ventricular

In conclusion, this study demonstrates a relevant additional diagnostic value of a pocket-size imaging device used in combination with the PE in a population of non-primary cardiologic patients. Pocket-size imaging devices can be very useful for detecting subclinical cardiac abnormalities in asymptomatic or pauci-symptomatic outpatients. Sensitivity and specificity are good in expert operators but suboptimal in trainees. Specificity is particularly influenced by the level of experience. Appropriate training programs are needed for all the potential users of pocket-size imaging devices.

## Competing interests

The authors declare that they have no competing interests

## Authors' contributions

MG conceived of the study and participated in its design and coordination, performed the statistical analysis and drafted the manuscript, AS participated in the study design and coordination and performed echo scans, MV, VSL and RE participated in the study coordination and performed echo scans, RR, RF, PLS, MB and MO participated in the study coordination and performed echo scans, GdS participated in the study design, performed and revised the statistical analysis and revised the manuscript. All authors read and approved the final manuscript.
